# The ISSPP PIPAC database: design, process, access, and first interim analysis

**DOI:** 10.1515/pp-2021-0108

**Published:** 2021-04-22

**Authors:** Michael Bau Mortensen, Olivier Glehen, Philipp Horvath, Martin Hübner, Kim Hyung-Ho, Alfred Königsrainer, Marc Pocard, Marc Andre Reymond, Jimmy So, Claus Wilki Fristrup

**Affiliations:** Department of Surgical Oncology, Centre Hospitalier Lyon Sud, Pierre Bénite, France; Department of General, Visceral and Transplant Surgery, University of Tübingen, Tübingen, Germany; Department of Visceral Surgery, Lausanne University Hospital (CHUV), University of Lausanne (UNIL), Lausanne, Switzerland; Department of Surgery, Seoul National University Bundang Hospital, Seongnam, Korea; Department of Surgery, Pitie Salpêtriére University Hospital, Paris, France; Department of Surgery, Yong Loo Lin School of Medicine, National University of Singapore, Singapore, Singapore; Department of Surgery, Odense University Hospital, Odense, Denmark; Department of Surgery, Odense PIPAC Center and Odense Patient data Explorative Network (OPEN), Odense University Hospital, Sdr. Boulevard, DK-5000Odense C, Denmark

**Keywords:** database, ISSPP, PIPAC

## Abstract

**Objectives:**

Several trials have documented the favorable safety profile, and promising clinical results of pressurized intraperitoneal aerosol chemotherapy (PIPAC) directed treatment in different types of peritoneal malignancies. However, until the results of randomized trials are available, the quality of documentation and acceptance by the users may be improved through a worldwide registry. The International Society for the Study of Pleura and Peritoneum (www.ISSPP.org) facilitated this process by creating a dedicated focus group and providing the funding needed for the creation and implementation of an international database. This article describes the design and the journey of establishing this international database and the first, preliminary results from the ISSPP PIPAC online database.

**Methods:**

In 2019 the ISSPP PIPAC Registry Group started to create a database with a minimal dataset relevant to many diseases and applicable in different framework conditions. The task was divided into three phases including design, testing, implementation, protocol, handbook, legal requirements, as well as registry rules and bylaws for the registry group.

**Results:**

The ISSPP PIPAC online database has six key elements (patient, consent, treatment, complications, response evaluation and follow-up). Following design, testing and implementation the database was successfully launched in June 2020. Ten institutions reported on 459 PIPAC procedures in 181 patients during the first 6 months, and the recorded data were comparable to the present literature.

**Conclusions:**

A new international multicenter PIPAC database has been developed, tested and implemented under the auspices of ISSPP. The database is accessible through the ISSPP website (www.ISSPP.org), and PIPAC institutions worldwide are highly encouraged to participate.

## Introduction

Pressurized intraperitoneal aerosol chemotherapy (PIPAC) is an innovative drug delivery technique designed to improve the effect of intraperitoneal administered chemotherapeutic agents [1]. Several phase I and II trials have documented the favorable safety profile, and promising clinical results of PIPAC directed treatment in different types of peritoneal malignancies [2], [3], [4], [5], [6], [7], [8]. However, until the results of randomized trials are available, PIPAC cannot be considered as a standard-of-care method [9], [10], [11]. PIPAC is a generic technique, and potential indications are numerous, including rare diseases. Well-known chemotherapeutic drugs are applied (cisplatin, oxaliplatin, doxorubicin, nab-paclitaxel, etc.), but none of these drugs is approved for intraperitoneal delivery. Hence, PIPAC remains so far an “off-label” therapy. Further challenges are occupational health safety, regulatory issues, reimbursement (in the absence of cost-effectiveness data), and the involvement of a wide range of dedicated specialists and institutions.

According to the IDEAL framework, PIPAC has now reached the exploration phase (Stage 2b) as the technique was well described and more centers have adopted this procedure. Prospective registry is recommended at this stage to document the safety and clinical outcomes from different centers for learning purpose [12]. Currently, most PIPAC procedures are performed in high volume, experienced cancer centers. Early in their experience, these centers expressed the wish to share data (in particular indications, number of procedures, technique, drug, dose, complications, etc.). In January 2016, they started a multicenter, international online documentation (registry) of indications and results of PIPAC and the IDEAL framework (PITAC) for treating malignant peritoneal and pleural diseases (www.clinicaltrials.gov, NCT03210298). This registry was closed in 2020 after reaching the number of patients planned. Results are currently being analyzed. Since many patients cannot be treated within the framework of controlled clinical studies, there was a need for a second registry building upon the experience gained. The aim was to improve further the quality of documentation and acceptance by the users. The International Society for the Study of Pleura and Peritoneum (www.ISSPP.org) facilitated this process by creating a dedicated focus group and providing the funding needed. This article presents the results of this work and the first, preliminary results from the ISSPP PIPAC online database, which is now fully implemented and recruiting.

## Materials and methods

The ISSPP PIPAC database aims to monitor PIPAC activities and results worldwide, and for this purpose, the database uses few but relevant variables. This concept should then provide the foundation on which the international PIPAC community would enhance and expand the international collaboration, development, and scientific approach to treating primary and secondary malignant peritoneal diseases. Specifications were easy online access, user-friendly interface, drop-down menus, relatively few and easy to understand relevant variables, low time requirements, and simple monitoring of incomplete input. Besides, the database’s technical support and organization should be accessible, transparent, embracing, and open for relevant changes and potential new variables. Finally, an annual report should be provided to the participating centers.

### Ethical framework

The ISSPP PIPAC database and the study protocol were approved by the Region of Southern Denmark (GDPR, 20/18204) on April 21st, 2020; the Institutional Review Board of the University of Southern Denmark (SDU REC 20/24559) on May 5th, 2020 and by the hosting unit Odense Patient data Explorative Network (OPEN) on May 19th, 2020 (OP-1140, 19/49984).

### Software

The ISSPP PIPAC database was developed using the REDCap software. REDCap was originally created by the Vanderbilt University, Nashville, USA, to support the secure collection of clinical data. REDCap is used by numerous institutions and researchers worldwide. It has been maintained by the REDCap Consortium since 2006 and is available free of charge for nonprofit-organizations (www.project-redcap.org).

### Hosting

The ISSPP PIPAC database is hosted by OPEN (www.open.rsyd.dk). OPEN is part of an integrated research infrastructure supported by Odense University Hospital and the University of Southern Denmark, Odense, Denmark. Database management, maintenance and monitoring are performed by OPEN in close collaboration with the ISSPP PIPAC database Manager (Research Project Manager, RPM, Dr. Claus Wilki Fristrup), the ISSPP Registry Group chairman (Professor Michael Bau Mortensen), and the ISSPP Registry Group.

### Governance

The PIPAC registry is a proprietary project of the International Society for the Study of Pleura and Peritoneum (www.isspp.org), a nonprofit organization registered at the Administrative Court in Stuttgart, Germany, under the number (VR 724228), with the tax number (Finanzamt Tübingen). 86166/557789. Its Executive Committee legally represents ISSPP.

### Methodology of implementation

The ISSPP registry group decided to focus on two major tasks:

1) Design, testing and implementation of the new database
2) Create documentation, including registry handbook, standards of practice (SOPs), registry rules and bylaws for the steering committee.

These tasks were completed between November 2019 and April 2020 and coordinated by the Odense PIPAC Center, Denmark. There were three phases in project management.

– **Phase I** (November 2019 – March 2020)

Phase I included the creation, discussion, and correction of the following documents:

– The patient consent form,
– Patient module,
– Treatment module,
– Complications module,
– Follow up module, including tumor response.

These documents were first drafted during the First Scientific Congress of ISSPP which was held in Singapore in November 2019. After the programming work, the new PIPAC online database was first tested locally at Odense PIPAC Center, Denmark, and several corrections were implemented. Then, a beta version of the database was tested by a handful of international centers, and the second round of edits was completed. The database guidelines, annual database report design, and bylaws were generated and submitted to internal review in parallel.

– **Phase II** (April 2020–August 2020)

The ISSPP PIPAC database was launched in June 2020. During Phase II, the main activities were inclusion from new institutions, monitoring and correction of errors based on feedback from reporting centers. In parallel, we started a prospective quality control of the data entered, including the listing of incomplete data entries for each center. We performed a first exploratory analysis to test the ability of the database to deliver the information expected.

– **Phase III** (September 2020–August 2021)

Phase III is currently ongoing and includes a regular reminder of incomplete data entries, monitoring participating centers and data collection, data analysis, and drafting the first annual report. An annual revision of the dataset is scheduled. Besides the annual scientific report, the ISSPP Registry Group will report to ISSPP Executive Committee on the project progress, the legal and financial aspects. Finally, the ISSPP Registry Group will report on its activities in the society’s peer-reviewed journal.

### Financial aspects

The PIPAC database is funded by an annual grant of ISSPP and by OPEN, Odense University Hospital, Odense, Denmark, which is hosting and maintaining the database free of charge. The inclusion of new centers, change of variables, providing and presenting data for the annual report to the ISSPP Registry Group, are performed by the RPM.

## Results

The PIPAC database was launched on June 15th, 2020, on the official ISSPP website (www.isspp.org). The access is password protected. The launch was prepared in detail, starting with local testing at Odense PIPAC Center in January 2020. Then, the beta version of the database underwent international testing in five centers (Lausanne, Switzerland; Lyon and Paris, France; Singapore; Tübingen, Germany) in February 2020. The documents were evaluated by the test centers, and after a few edits, they were sent by the ISSPP Registry Group to ISSPP’s executive committee for approval, together with the bylaws and an interim progress report. ISSPP’s Executive Committee provided its legal consent early in April 2020, but the launch was only effective by June 15th, 2020, due to the Covid-19 pandemic.

### Legal aspects

The database meets the requirements of the European General Data Protection Regulation (GDPR). Pseudo-anonymized data are transferred to OPEN based on the patient consent obtained in each center. The registry data do not leave European soil. For participating centers, legal issues may vary across countries. Still, the minimal universal requirement is the patient signature on the patient consent form and the nondisclosure of identifying personal information. Acquiring the patient signature and compliance with local regulations are the responsibility of each participating center, including non-EU centers.

### Access to the ISSPP PIPAC database

Access to the database is password-protected. Access requirements are an active ISSPP membership and registration with the database. ISSPP members can find a user request form for the database on the society’s website. Users are required to fill out the form and mail it to the database manager. The final account creation requires a manual process, and some waiting time may occur after submitting the request by e-mail. Once the registration process is completed the database can be accessed at https://open.rsyd.dk/redcap/. More information can be found on the ISSPP website (http://isspp.org/professionals/pipac-database/).

### Database modules and components

The goal of the first iteration of the database was to keep the number of variables low in order to facilitate a quick data entry process for the PIPAC procedures (Table 1). The emphasis is on getting a high degree of complete and valid data entries. The consent form documents the patients’ informed consent to participating in the ISSPP PIPAC database. The patient module is the primary entry form for the database; it holds data on demography, primary tumor, and treatment before PIPAC. The number of PIPAC procedures for each patient is not limited. The Treatment, Response, and Complications modules are used for each PIPAC procedure. Complications are graded according to either Dindo-Clavien (surgical complications) or the Common Terminology Criteria for Adverse Events (CTCAE v. 5, for medical complications). The follow-up module holds information on the last contact (e.g., date of death) and reason(s) for stopping additional PIPAC directed therapy. Response evaluations are registered using either the peritoneal regression grading score [13] or merely reporting the level of response. Complete documentation of the different modules and variables can be found in the codebook on the ISSPP website. The current REDCap data dictionary is published there and allows participating institutions to host and extend a copy of the database – limited to their own cases.

**Table 1:** Variables of the ISSPP PIPAC database.

Patient	Treatment
Local ID	Date of treatment
Date of birth	Date of discharge
Gender	Treatment within prospective study
Primary tumor	Electrostatic precipitation
Primary tumor *in situ*	Other surgical procedures
Primary histology	Drug – type of drug and dosage
Date of diagnose of peritoneal metastasis	Flow rate of infusion
Time from primary tumor diagnosis to peritoneal metastasis	Exposure time (min)
Metastasis outside peritoneum	PCI score
Location of extraperitoneal metastasis	Histological response evaluation performed
Previous oncological treatment	Ascites
ECOG performance status	Systemic chemotherapy within 4 weeks prior to this treatment
	Complications – details registered separately

**Response evaluation**	**Complications**

Date of biopsy	Date of complication/Adverse event
Type of evaluation	Complication/Adverse event (CTCAE v5.0)
Mean peritoneal regression grading score (PRGS) Non PRGS evaluation	Either: CTCAE grade or Dindo-Clavien Grade

**Follow up**	

Date of latest follow up dead – date of death	
Reasons for stopping PIPAC	

### Handling of errors/missing values

The proportion of missing values was low (<5%) for the majority of variables. The primary type of error was an erroneous entry in the calculation of age and length of stay. These data were either excluded (age<20 or >120 years, length of stay<0 days) or handled by using median values (in the case of an erroneous length of stay).

### Patient recruitment and first interim analysis

Six months after the launch, 10 institutions from around the world have joined the database. There was a steady increase in the number of cases. Currently (as of December 29th, 2020), the database contains data on 181 patients and 459 PIPAC procedures. Forty-four (24%) of the patients were treated with PIPAC-directed therapy within prospective clincal trials. Demographic data are listed in Table 2. The included patients had median 2 PIPAC procedures (range 1–8) with a nonaccess rate of 13% at the first PIPAC (Table 3). Only one grade 3 surgical complication was registered in the 459 procedures (0.2%). Adverse events according to CTCAE were all grade 1 and 2 (Table 4).

**Table 2:** Demographic data from patients included between June 2020 and December 2020.

Variable	Value
No. Patients	181
Age, years (range)	58 (28–82)
Female/male	97/84
ECOG performance status	0: 12.2%
	1: 82.3%
	2: 4.4%
	N.A. 1.1%
Primary tumor	n
Gastric	56
Colon	36
Appendix	25
Pancreas	17
Ovaries	11
Bile duct	9
Mesothelioma	6
Small bowel	3
Esophagus	2
Brest	2
Fallopian tube	1
Uterus	1
Primary peritoneal	1
Other	11
Primary tumor, *in situ*	66%
Histology, primary tumor	Adeno 39%
	Mucinous adeno 11%
	Signet-ring-cell carcinoma 20%
	Other 30%
Synchronous PM (0–2 months from diagnosis of primary tumor)	54%
Extraperitoneal metastases	5.6%
Previous oncological treatment	Yes 68.2%
	No 31.8%
Type of treatment	Systemic chemo 65.8%
	Immunotherapy 1.1%
	Radiotherapy 2.2%
	Other 9.4%

**Table 3:** Treatment.

Total number of procedures	459
Number of PIPAC per patient Median (range)	2 (range 1–8)
Median length of stay (95% percentile)	2 days (4 days)
Non-access at first PIPAC (overall non-access rate)	13% (7%)
Electrostatic precipitation	7%
Other surgical procedures	6 (1%)
PCI registration at first PIPAC (all procedures)	83% (76%)
PCI score at first PIPAC Median (range)	17 (0–39)
PCI score all procedures Median (range)	21 (0–39)
Use of histological response evaluation	Non-PRGS: 69% PRGS: 28% None: 3%
Ascites, reported cases and median volume (range)	n=211 200 mL (0–8,500 mL)
Use of systemic chemotherapy between PIPAC	24%

**Table 4:** Complications and adverse events.

Total number of procedures	459
Number of procedures with recorded complications	77 (19%)
Number of detailed and graded event	Surgical: 4 Adverse events: 129
Surgical complications	Grade 1: 3 (hematoma, bleeding, bowel injury) Grade 3: 1 (bowel injury)
Adverse events	Grade 1: 59 Grade 2: 70
Most common adverse events, n	Abdominal pain (43) Nausea (25) Other (15) Constipation (12) Vomiting (11) Urinary retention (10)

### Annual report

Once a year, the RPM will analyze the dataset. Results will be summarized and presented in the Annual ISSPP PIPAC database Report. Members of the ISSPP Registry Group will write the report draft, submitted to the legal entity (ISSPP) before publication. After approval, the results will be published in *Pleura & Peritoneum*, on the ISSPP website, and presented at the following international ISSPP Congress. Once a year, based on its own experience and the participating centers’ feedback, the ISSPP Registry Group will implement the changes needed.

## Discussion

Since 2011, when the first PIPAC directed treatment of peritoneal metastases in humans was performed [14], the number of procedures has steadily increased worldwide, exceeding 10,000 at the end of 2020 (Capnomed GmbH, Zimmern o.R., Germany, private communication). The evidence is multiplying with ongoing, and results from Phase-I [2–4, 15] and Phase-II [5], [6], [7], [8] trials published. Twenty-four clinical trials are currently listed at ClinicalTrials.gov (January 15, 2021), including two randomized Phase-2 trials [16], [17]. However, in the absence of controlled results comparing PIPAC vs. standard of care, PIPAC-directed treatment has not yet reached the level of evidence needed for full recognition within the oncology community [11]. The number of patients treated “off-label” as an individual therapy outside the framework of clinical studies, the procedure’s generic character, the numerous indications, and the multiple drugs available are arguments in favor of gathering additional data through a quality-controlled, international registry.

This article presents the development, testing, and implementation of the quality-controlled ISSPP PIPAC database as well as the preliminary data gathered between July 2020 and December 2020.

The ISSPP PIPAC database was successfully designed, tested and implemented using a stringent sequence of tasks and deadlines. Within six months, despite the Covid-19 pandemic, the database received the official approvals needed and went online. The users’ feedback is so far positive, and it seems possible to include patient data easily using drop-down menus and within a reasonable time frame—although this was not yet evaluated in detail.

The first preliminary analysis illustrates that the patients who entered into the ISSPP PIPAC Database mirror the general experience with PIPAC as presented in the literature (e.g. primary diagnosis, number/type of procedures, and complications) [10]. It is important to note that the data have been entered by a few centers with variable inclusion rates. However, the preliminary results present selected data to illustrate the additional value of such registry. For example, data on PIPAC in rare indications have been collected from individual institutions, which experience is now merged.

A successful clinical database is characterized by clinically relevant questions and variables, easy access, low time requirements, high participation and data completeness rates. In addition, the organization behind the database (i.e. the ISSPP Registry Group) should be transparent, embracing, and open for relevant changes. The database should be dynamic and enable updates based on conclusions drawn from regular outcome reports.

The initial step was not to create a research database but to set up a registry to monitor PIPAC activities and results worldwide. Now we will actively promote the inclusion of additional PIPAC centers and create the first annual ISSPP PIPAC database report in 2021. Simultaneously, the ISSPP PIPAC Registry Group will start to include data from other institutional and national databases (Phase IV) and merge data on rare peritoneal diseases treated by PIPAC (e.g. Malignant Peritoneal Mesothelioma).

## Conclusions

A new international multicenter PIPAC registry has been developed, tested and implemented under the auspices and the legal responsibility of ISSPP, which ensures transparent, independent governance and compliance with good scientific practice rules. The database is accessible through the ISSPP website (www.ISSPP.org), and PIPAC institutions worldwide are highly encouraged to participate.
